# Acute Macular Neuroretinopathy Associated With COVID-19 Infection: Is Double Heterozygous Methylenetetrahydrofolate Reductase (MTHFR) Mutation an Underlying Risk Factor?

**DOI:** 10.7759/cureus.34873

**Published:** 2023-02-11

**Authors:** Christina Karakosta, Evgenia Kontou, Tina Xirou, Stamatina A Kabanarou

**Affiliations:** 1 Ophthalmology, Korgialenio-Benakio Hellenic Red Cross Hospital, Athens, GRC

**Keywords:** maculopathy, covid-19, homocysteine levels, viral infection, macular ischemia

## Abstract

The goal of this report is to present a case of coronavirus disease 2019 (COVID-19)-associated acute macular neuroretinopathy (AMN) with an underlying *MTHFR *mutation.

A 36-year-old male presented to the emergency department with a sudden-onset paracentral scotoma in his left eye. Although optical coherence tomography (OCT) was normal initially, four days later, it revealed a hyperreflective band in the outer plexiform layer with disruption of the ellipsoid zone/interdigitation zone. On infrared imaging and en-face OCT, wedge-shaped lesions were detected around the fovea with their tip oriented toward the fovea. OCT angiography, fundus autofluorescence, fundus fluorescein angiography, and visual fields were performed. The patient was positive for COVID-19 infection. The absence of medical history and the negative results of blood tests led to a diagnosis of AMN associated with COVID-19. Genetic testing for coagulation disorders was scheduled and revealed a heterozygous mutation for *MTHFR C677T* and *A1298C*.

This is the first case of AMN in a patient with COVID-19 infection and a double heterozygous mutation of the *MTHFR* gene. Infection is the most commonly reported association of AMN, while *MTHFR *mutation may represent an additional underlying risk factor. Microthrombosis and small-vessel occlusion are thought to be involved in the pathophysiology of AMN, and patients should be tested for COVID-19 because it may be the first manifestation of the infection.

## Introduction

Acute macular neuroretinopathy (AMN) is a rare condition first reported in 1975 by Bos and Deutman [1]. Its primary feature is the acute onset of a paracentral scotoma, and it mainly affects young healthy women [2]. On near-infrared imaging, reddish-brown teardrop or wedge-shaped lesions are detected around the fovea with their tip oriented toward the fovea [3]. These lesions, which correspond to the scotoma described by the patient, may not be visible on fundoscopy. The lesions typically resolve over time, in contrast to scotomas, which persist [2]. Retinal vessels and optic nerve are normal, and vitreous inflammation is absent.

Although the pathogenesis of AMN remains unclear, data suggest that ischemia of the deep capillary plexus of the central retina occurs [4-6]. Another hypothesis is that microthrombosis and small-vessel occlusion occurs in the context of inflammation, including severe acute respiratory syndrome coronavirus 2 (SARS-CoV-2) virus infection and vaccination [7,8]. Optical coherence tomography (OCT) examination reveals two distinct patterns of AMN. In type 1, the middle retina or inner nuclear layer (INL) is affected and a hyperreflective band is seen superficial to the outer nuclear layer (ONL). In type 2, the outer retina is affected, including both the ONL and the ellipsoid zone, and a hyperreflective band is seen deep to the outer plexiform layer (OPL) with disruption of the ellipsoid zone/interdigitation zone [3,5,6]. On OCT angiography, reduced flow signals in deep retinal capillary plexus are noted. Fundus fluorescein angiography (FFA), indocyanine green angiography, and fundus autofluorescence (FAF) are usually normal.

Infection is the most commonly reported association of AMN. Other associations include oral contraceptive pills, significant coffee consumption, use of epinephrine, hypotensive episodes, and recent vaccination [2]. Coronavirus disease 2019 (COVID-19) infection and vaccination have been recently described as risk factors of AMN [9,10]. Here, a case of COVID-19 infection-associated AMN is presented using multimodal imaging.

## Case presentation

**Figure 1:**
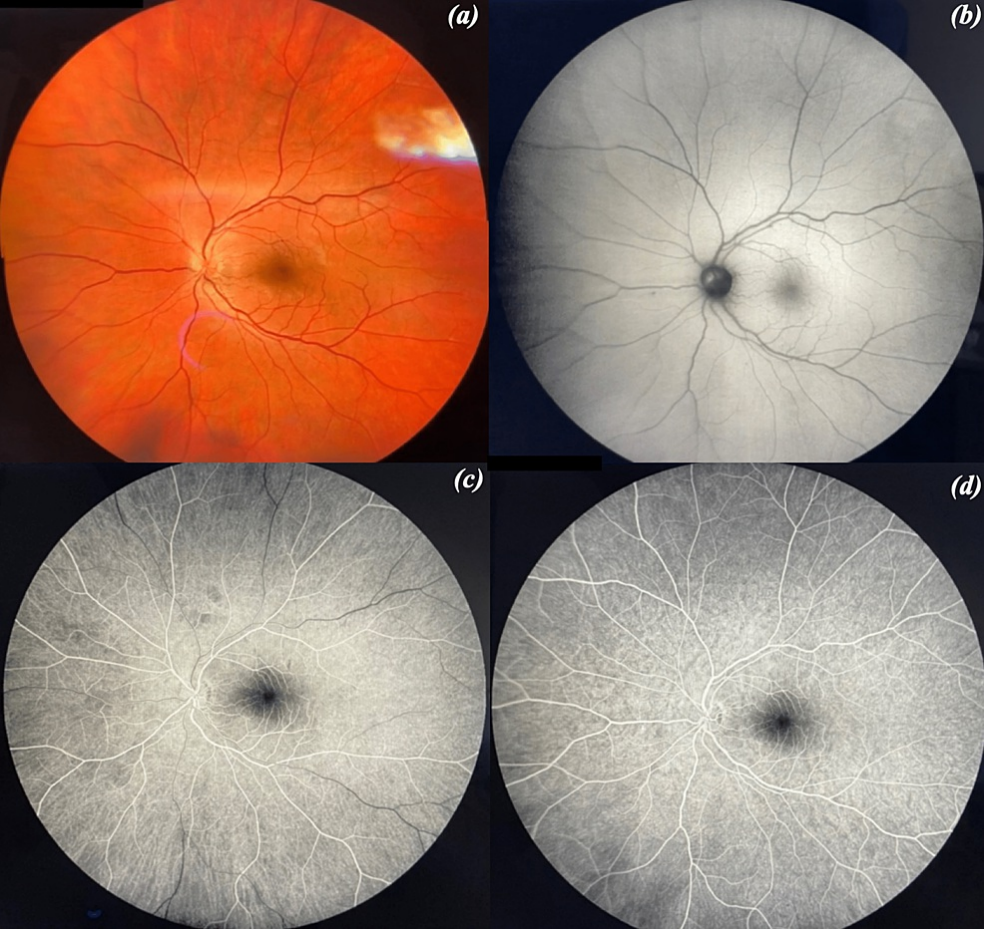
(a) Fundus of the OS showing the blurry margin of the optic disc. (b) Fundus autofluorescence of the OS showing optic disc drusen. (c) Fundus fluorescein angiography of the OS - early phase. (d) Fundus fluorescein angiography of the OS - late phase. OD = right eye; OS = left eye

A 36-year-old Caucasian male, a former smoker with no medical history, presented to the emergency department with a sudden-onset paracentral scotoma, which started two hours ago in his left eye (OS). The examination showed that his best-corrected visual acuity (BCVA) was 20/20 in his right eye (OD) and 20/20 in OS. No relative afferent pupillary defect (RAPD) was present. The Ishihara test was normal in both eyes (14/14 Ishihara plates in both eyes). Slit-lamp biomicroscopy was normal in both eyes. Intraocular pressure was within normal limits (14 mmHg in OD and 15 mmHg in OS). Fundoscopy was normal in OD, while it revealed blurry optic disc margins in OS (Figure 1, Panel a).

**Figure 2:**
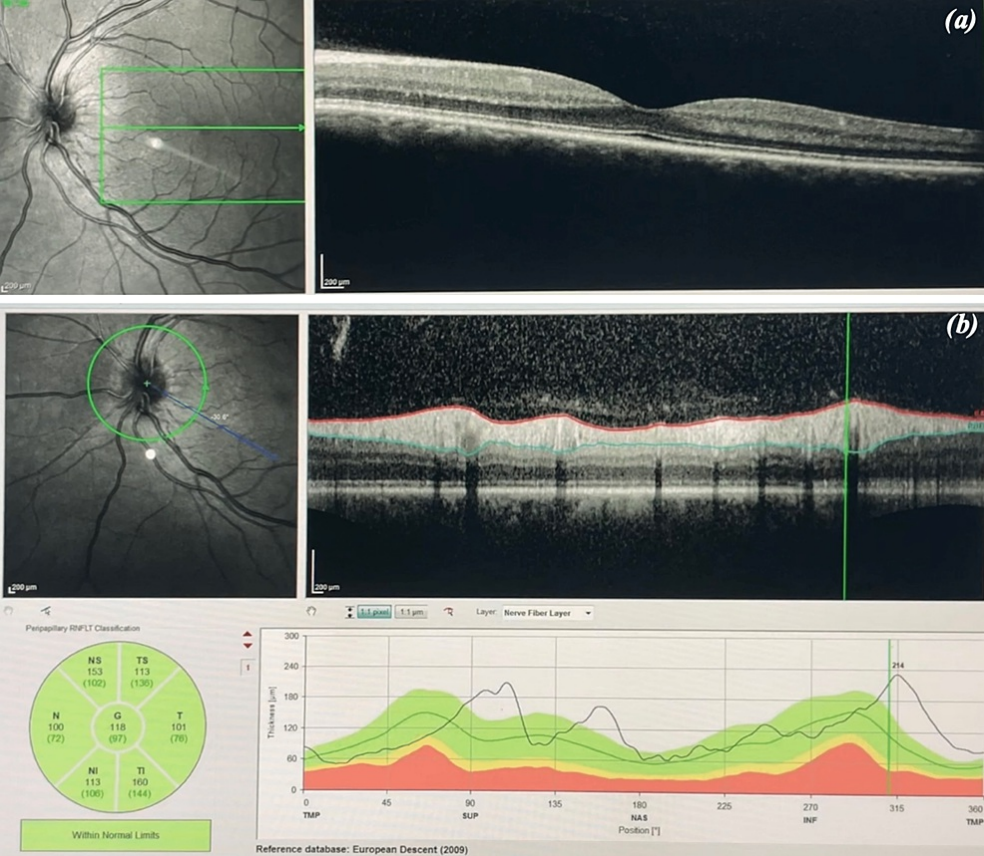
(a) OCT of the macula of the OS within normal limits on day one. (b) OCT of the OS showing RNFL thickening on day one. OCT = optical coherence tomography; OD = right eye; OS = left eye; RNFL = retinal nerve fiber layer

Macular OCT was normal in both eyes (Figure 2a). OCT of the retinal nerve fiber layer (RNFL) revealed thickening in the OS (Figure 2b), while it was normal in the OD.

FAF showed optic disc drusen in the OS (Figure 1, Panel b), which was confirmed with B-scan ultrasonography.

**Figure 3:**
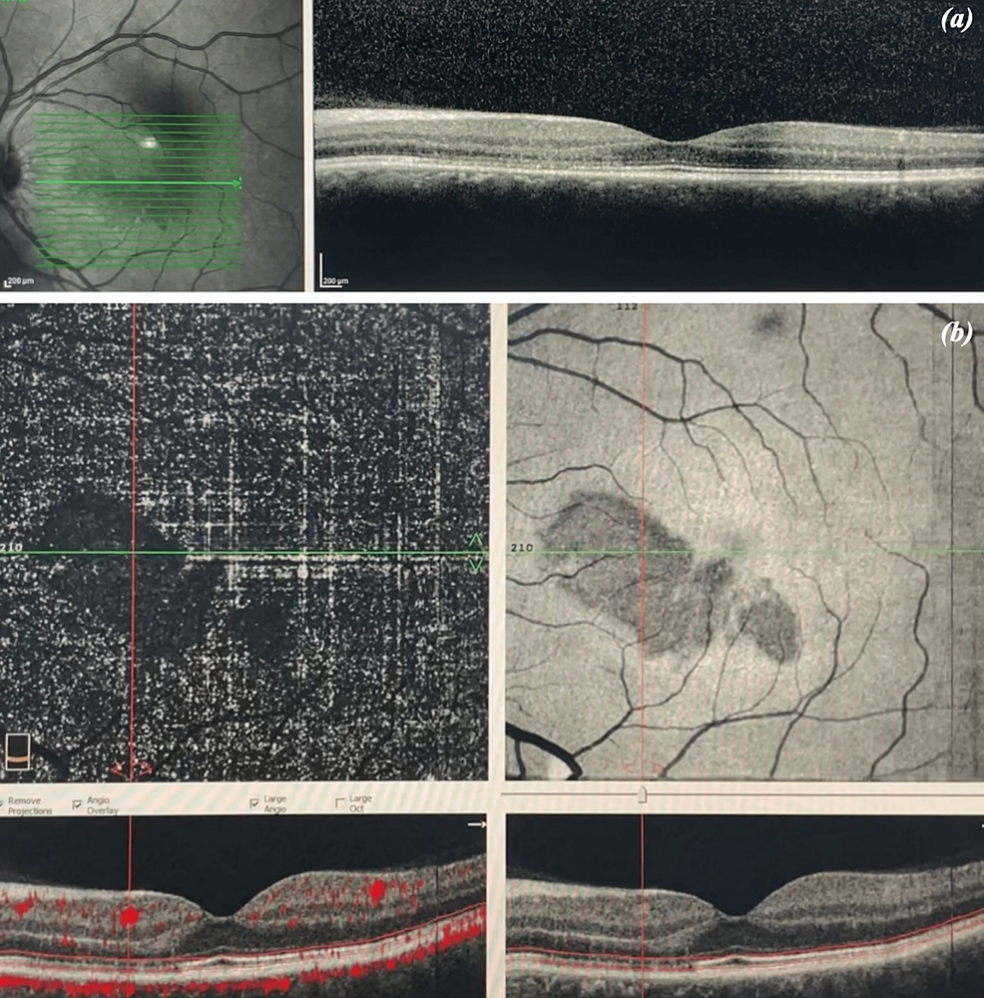
(a) OCT of the macula of the OS showing a hyperreflective band in the outer plexiform layer with disruption of the ellipsoid zone/interdigitation zone on day four. (b) En-face OCT of the macula of the OS (top right) showing wedge-shaped lesions around the fovea with their tip oriented toward the fovea. OCT = optical coherence tomography; OS = left eye

Visual fields were performed (24-2 and 10-2), which were normal in the OD, while a superior/temporal paracentral scotoma was revealed in the OS. Blood tests including full blood count, C-reactive protein, erythrocyte sedimentation rate, international normalized ratio, prothrombin time, activated partial thromboplastin time, and lipid profile were normal. Homocysteine levels were normal as well. The patient suffered from flu-like symptoms, including sore throat, nasal congestion, and low fever which started a few days ago. He was fully vaccinated against COVID-19. A real-time reverse transcription-polymerase chain reaction (RT-PCR) test was performed and the result was positive for SARS-CoV-2. His blood pressure was 126/73 mmHg, his heart rate was 75 beats per minute, his body temperature was 36.9°C, and his blood oxygen saturation was 99%. A triplex of the carotid arteries was scheduled which was within normal limits. The patient was reevaluated in our clinic four days later. His BCVA was 20/20 in both eyes. Fundoscopy did not show any changes in both eyes. However, OCT revealed a hyperreflective band in the OPL with disruption of the ellipsoid zone/interdigitation zone (Figure 3a). On infrared imaging (Figure 3a) and on en-face OCT (Figure 3b), wedge-shaped lesions were detected around the fovea with their tip oriented toward the fovea.

**Figure 4:**
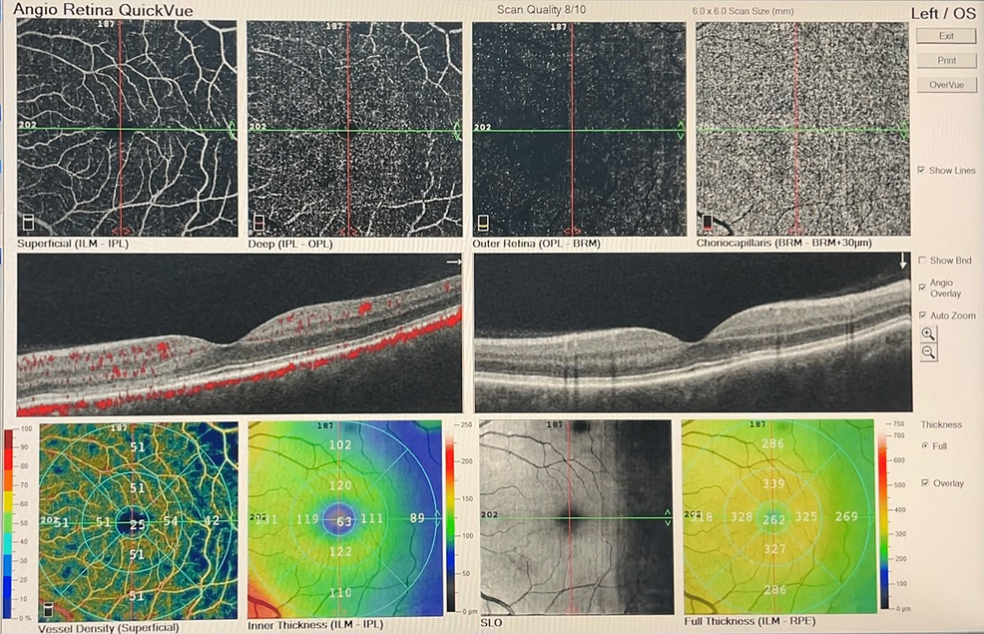
OCT-A of the OS showing reduced flow signals in the deep retinal capillary plexus on day four. OCT-A = optical coherence tomography angiography; OS = left eye

OCT-A revealed reduced flow signals in the deep retinal capillary plexus (Figure 4).

**Figure 5:**
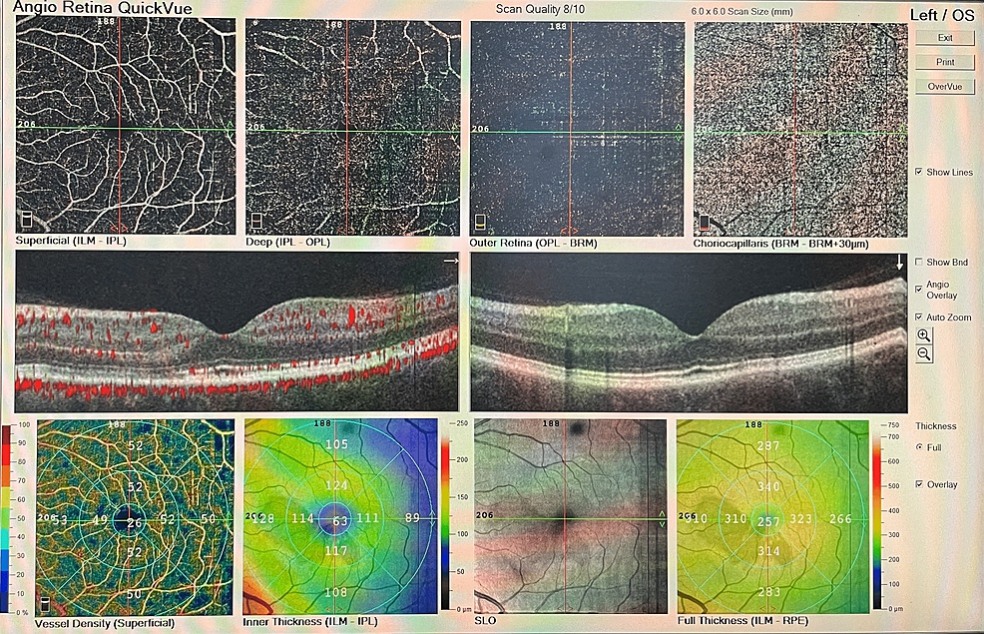
OCT-A of the OS on day seven. OCT-A = optical coherence tomography angiography; OS = left eye

Based on the patient’s history and ocular assessments, a diagnosis of AMN associated with COVID-19 infection was made. FFA was scheduled seven days later, which was normal in the OS (Figure 1, Panels c, d), and OCT-A was performed again (Figure 5).

**Figure 6:**
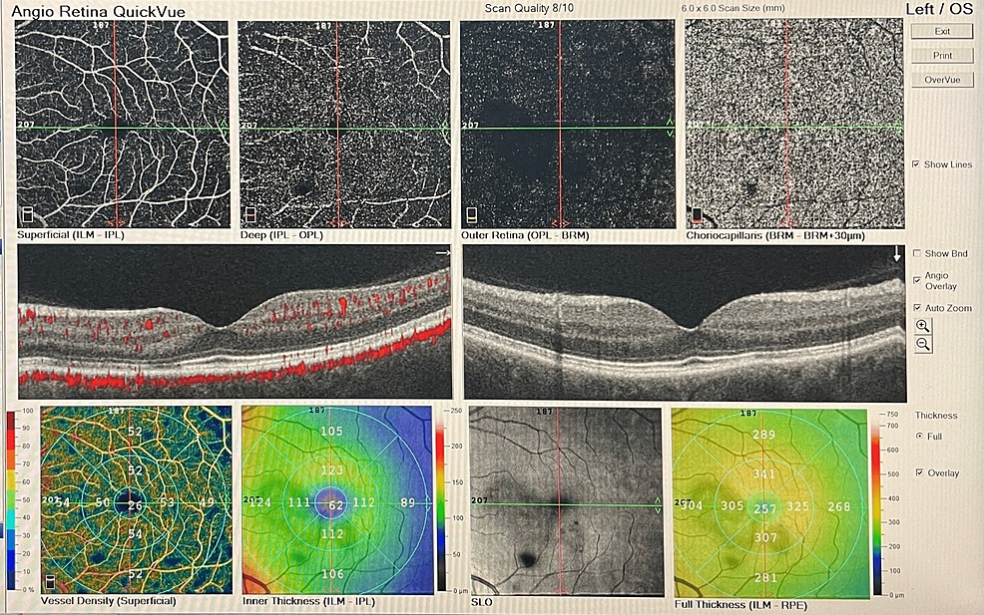
OCT-A of the OS one month later. OCT-A = optical coherence tomography angiography; OS = left eye

Genetic testing for coagulation disorders was scheduled and revealed a heterozygous mutation for *MTHFR C677T* and *A1298C*. The patient was reevaluated in our clinic one and six months later. On the OCT scan, disruption of normal retinal banding patterns was present at the junction of OPL-ONL and on the ellipsoid zone in the OS, while on OCT-A, hypoperfusion was noted at the level of the deep vascular complex (Figure 6).

**Figure 7:**
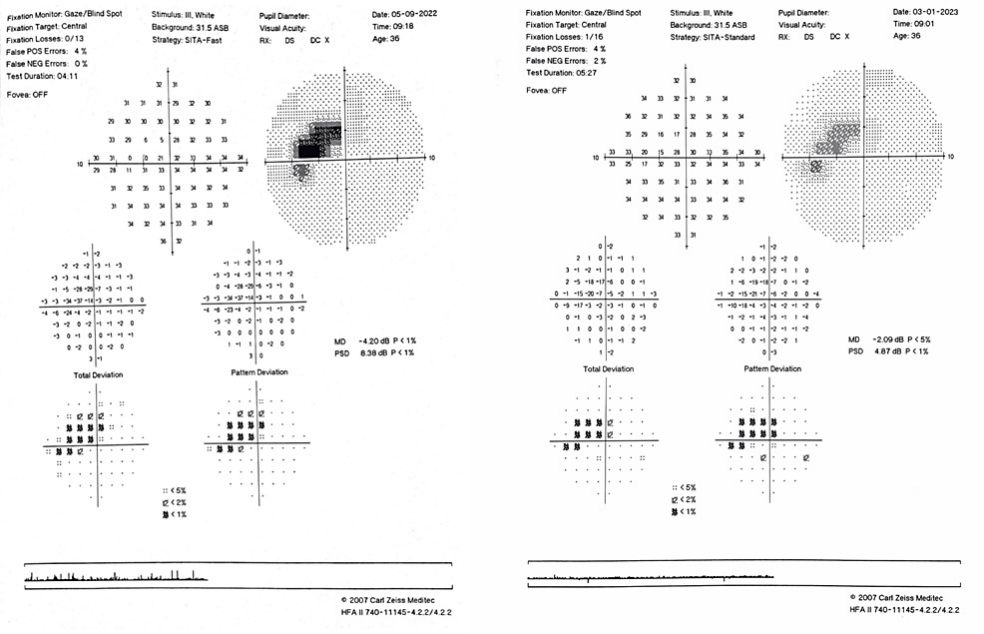
Visual field test (10-2) of the OS one month (left image) and six months (right image) after the onset of AMN, showing the persistent paracentral scotoma. OS = left eye; AMN = acute macular neuroretinopathy

A visual field examination (10-2) showed persistent paracentral scotoma (Figure 7).

A Mediterranean diet and annual assessment of serum homocysteine levels were recommended based on the genetic testing results.

## Discussion

MTHFR (methylenetetrahydrofolate reductase) is an enzyme necessary for the metabolism of methionine and the formation and recycling of homocysteine. Mutations in the *MTHFR *gene affect the activity of the enzyme, leading to elevated homocysteine levels. Common mutations include *C677T *and *A1298C *[11]. Double heterozygous refers to patients who have heterozygous mutations for both of these mutations [12]. Hyperhomocysteinemia is a risk factor for vascular thrombosis, causing a prothrombotic state through oxidative stress and endothelial dysfunction [13]. The presence of *MTHFR *gene mutation has been reported to be associated with retinal vascular occlusion in the past [13], but this is the first case of AMN in a patient with COVID-19 infection and a double heterozygous mutation of the *MTHFR* gene.

Viral flu-like illnesses and vaccines have already been associated with AMN. COVID-19 infection causes a pro-inflammatory and hypercoagulable state, leading to multiple systemic complications, primarily, respiratory and cardiovascular [14]. Various ocular manifestations have been reported due to COVID-19 infection, with conjunctivitis being the most common [14]. An increase in retinal vascular manifestations due to COVID-19 infection has been reported as well, including AMN [7]. To date, there have been several cases of AMN secondary to COVID-19 infection. Although the pathogenesis of AMN, and particularly of AMN secondary to COVID-19 infection, is unclear, it may be explained by immune-mediated tissue damage, activation of the coagulation cascade, and prothrombotic state induced by a viral infection, which leads to microthrombosis and small-vessel occlusion, and, thus, deprivation of oxygen and nutrients and ischemia of the deep capillary plexus of the central retina [14]. In particular, microvascular ischemia of the choriocapillaris causes hypoxia of the middle and outer retinal layers in combination with vasoconstriction due to cell-mediated stressors [9].

## Conclusions

AMN may be an ocular manifestation secondary to COVID-19 infection. *MTHFR *mutation may be an underlying risk factor for AMN in these patients. The exact mechanism remains unclear, but it probably includes coagulation anomalies and hyperinflammation. Patients diagnosed with AMN should be tested for COVID-19 because AMN may be the first manifestation of the infection, and genetic testing should be scheduled. The association between AMN and double heterozygous *MTHFR *mutation may be investigated in future studies.
